# Enhancing the mechanical properties and providing bioactive potential for graphene oxide/montmorillonite hybrid dental resin composites

**DOI:** 10.1038/s41598-022-13766-1

**Published:** 2022-06-17

**Authors:** Marilia Mattar de Amôedo Campos Velo, Francisco Gilmário Nunes Filho, Tatiana Rita de Lima Nascimento, Alyssa Teixeira Obeid, Lúcio Cançado Castellano, Reginaldo Mendonça Costa, Nair Cristina Margarido Brondino, Maria Gardennia Fonseca, Nikolaos Silikas, Rafael Francisco Lia Mondelli

**Affiliations:** 1 Department of Dentistry, Endodontics and Dental Materials, Bauru School of Dentistry, University of São Paulo, Al. Dr Octávio Pinheiro Brisolla, 9-75, Bauru, São Paulo 17012-901 Brazil; 2 Department of Chemistry, Research and Extension Center for Fuels and Materials Laboratory (NPELACOM), Federal University of Paraiba, João Pessoa, Paraíba Brazil; 3 Human Immunology Research and Education Group (GEPIH), UFPB Technical School of Health, Federal University of Paraíba, João Pessoa, Paraíba Brazil; 4 Department of Mathematics, School of Sciences, São Paulo State University-UNESP, Bauru, São Paulo Brazil; 5 Dentistry, School of Medical Sciences, The University of Manchester, Manchester, M13 9PL UK

**Keywords:** Medical research, Biomaterials

## Abstract

This in vitro study synthetized hybrid composite nanoparticles of graphene oxide (GO) and montmorillonite MMt (GO-MMt) by ultrasound treatments. Samples were characterized by X-ray diffraction, FT-Raman, FTIR, TEM and SEM. The effect of their incorporation (0.3% and 0.5%) on the mechanical properties in a resin-based composite (RBC) and their bioactivity potential were evaluated. The specimens were characterized by evaluating their 3-point flexural strength (n = 6), modulus of elasticity (n = 6), degree of conversion (n = 6), microhardness (n = 6), contact angle (n = 3) and SEM analysis (n = 3). In vitro test in SBF were conducted in the RBCs modified by the hybrid. Overall, the synthetized hybrid composite demonstrated that GO was intercalated with MMt, showing a more stable compound. ANOVA and Tukey test showed that RBC + 0.3% GO-MMt demonstrated superior values of flexural strength, followed by RBC + 0.5% GO-MMt (*p* < 0.05) and both materials showed higher values of microhardness. All groups presented a contact angle below 90°, characterizing hydrophilic materials. RBCs modified by the hybrid showed Ca and P deposition after 14 days in SBF. In conclusion, RBCs composed by the hybrid showed promising results in terms of mechanical properties and bioactive potential, extending the application of GO in dental materials.

## Introduction

Over the years, several areas of engineering have combined different properties of materials to provide enhanced properties for an application. Such a strategy has been extrapolated to the biomedical field to improve properties, such as resistance, biocompatibility, bioactivity and aesthetics, to meet the patient demands. Since a single material that has all of the above properties is impractical, the development of hybrid composites improves the performance of individual components^1^. Fillers in a composite act as reinforcement and improve the mechanical properties or bioactivity of a material^2^.

In dentistry, the mechanical properties of resin-based composites (RBCs) have been constantly improved since they are related to their clinical application and the longevity of the restoration. Even though the incorporation of nanostructures improves the mechanical properties of RBCs, the popularity of RBCs is due to all their features, i.e., biocompatibility and aesthetic and mechanical properties^3,4^. The development of bioactive RBCs has also been investigated since they could prevent secondary caries, which has been recognized as one of the major causes for failure of restorations with resin composites^5^. Therefore, the synthesis of a hybrid composite that aggregates all these properties would be highly desirable and improve their clinical performance.

As successfully prepared by Geim and Novoselov in 2004^6^, graphene, an allotrope of carbon, presents remarkable physical properties, such as conductivity and mechanical stability^7^, high aspect ratio and low density, which make it the ideal candidate for developing the next generation of polymer composites^8^. Graphene oxide (GO) is a biocompatible material derivative of graphene modified by oxygen-containing groups that can be safely incorporated into scaffolds or nanocomposites to improve its mechanical properties for biomedical applications. The potent mechanism of antimicrobial action of GO has also been highlighted: GO mechanically disrupts bacterial membranes, leading to cell death, and even acts as a platform for the safe delivery of interappointment antimicrobial medicaments^9,10^. In addition, it is well known that GO particles present the ability to induce stem cell osteogenesis similar to that of bone morphogenic protein (BMP-2) and other bioactive inorganic materials, such as hydroxyapatite, which promotes nucleation and crystallization for the rapid hydroxyapatite growth high calcium–phosphate ratio in biomimetic conditions^11^.

Various studies have shown the influence of GO on the mechanical properties of conventional materials in dentistry, such as RBCs^12^, silane primers^13^, and adhesives^14^. GO was used to develop biocompatible dental materials for bone regeneration^15^, to protect against dental caries on the tooth root^16^ and to develop a composite with improved corrosion resistance and adhesion strength to titanium sheets^17^. In general, it has been suggested that the incorporation of a small amount of graphene can significantly improve a polymer's properties, especially the mechanical properties^18^. In addition, graphene presents some advantages over carbon nanotubes, such as lower cost, larger surface area, functional groups on its surface, and it does not increase the viscosity of the resin^19^. However, one of the main drawbacks of graphene to be applied as a reinforcement is the agglomeration that occurs in the matrix, which represents a challenge. To improve the adhesion between graphene and the matrix to achieve enhanced mechanical properties, the surface modification of graphene, i.e., using silane coupling agents, has been applied^20^. In a previous study, the incorporation of silane-modified graphene as a reinforcing phase was demonstrated to be an effective strategy for enhancing the tribological response of epoxy-based specimens^21^. The functionalization of the GO surface with γ-aminopropyltriethoxysilane and aminoacetic acid also provided a chemical interaction at the polymer matrix–filler interface, resulting in the strengthening of epoxy composites^20^.

Another method to overcome this issue is the combination of GO with other materials, as previously demonstrated in the synthesis of GO–zirconia or clays^12,20^. Some clay minerals, such as montmorillonite (MMt), which consists of aluminosilicate minerals, have emerged as attractive candidates for fixation on the surface of GO. The hydrophilicity, swelling properties and cation exchange capacity of MMt have positive effects on its adsorption performance^22^. Furthermore, mineral clay minerals can form electrostatic interactions and hydrogen bonds with GO layers, which allows the formation of hybrid materials or the preparation of suspensions of GO and MMt with good dispersion^22^. In GO and MMt hybrids, commonly formed by intercalation and costacking processes, the MMt phyllosilicate matrix provides stability to the carbonaceous layer structure, in addition to improving the physicochemical properties and biocompatibility, which could overcome some of the inconveniences of GO in dental materials^23^.

Thus, the present in vitro study synthetized hybrid composite GO nanoparticles functionalized with MMt (GO–MMt) and characterized their composition and morphology. The effect of their incorporation on the overall mechanical properties in an experimental resin matrix and their bioactivity potential were evaluated. It was hypothesized that the inclusion of GO–MMt within the dental resin matrix would present better mechanical properties than GO or MMt alone, as well as the bioactivity potential.

## Materials and methods

### Materials

The MMt used in the present work was purchased from Bentonisa do Nordeste SA, had a cation exchange capacity equivalent to 74.6 cmol (+)/kg and had the following chemical composition: SiO_2_ (52.98%), Al_2_O_3_ (18, 35%), Fe_2_O_3_ (3.96%), TiO_2_ (0.18%), CaO (0.01%), MgO (2.47%), Na_2_O (2.56%) and K_2_O (0.22%). GO, C_x_O_y_H_z_ (MM = 4239.48 g/mol) with a purity of 99% was purchased from Sigma–Aldrich.

The composition of the experimental RBCs (resin matrix) developed was composed of 49.5% BisGMA, 49.5% TEGDMA, 2-dimethacrylate 0.8% and camphorquinone 0.2% and is described in Table 1.

**Table 1:** Chemical composition of the experimental RBCs.

Material	Matrix monomers	Filler
G1 (control)	BisGMA, TEGDMA, 2-dimethacrylate and camphorquinone	–
G2 (MMt/0.3)	BisGMA, TEGDMA, 2-dimethacrylate and camphorquinone	0.3 wt.% MMt
G3 (MMt/0.5)	BisGMA, TEGDMA, 2-dimethacrylate and camphorquinone	0.5 wt.% MMt
G4 (GO/0.3)	BisGMA, TEGDMA, 2-dimethacrylate and camphorquinone	0.3 wt.% GO
G5 (GO/0.5)	BisGMA, TEGDMA, 2-dimethacrylate and camphorquinone	0.5 wt.% GO
G6 (GO–MMt/0.3)	BisGMA, TEGDMA, 2-dimethacrylate and camphorquinone	0.3 wt.% GO–MMt
G7 (GO–MMt/0.5)	BisGMA, TEGDMA, 2-dimethacrylate and camphorquinone	0.5 wt.% GO–MMt

*MMt*  montmorillonite, *GO*  graphene oxide, *Bis-GMA*  bisphenol A-glycidyl methacrylate, *TEGDMA*  triethylene glycol-dimethacrylate.

### Synthesis of graphene–montmorillonite (GO–MMt) hybrid composite

The GO–MMt mixture was prepared by ultrasound treatment and direct contact of aqueous suspensions of GO and MMt. Thus, a suspension of 4 g of MMt in 200 mL of water was sonicated with an ultrasonic probe (20 Hz, 50 W, amplitude 40) for 4 h. Suspensions of GO (20 mg and 40 mg) in 200 mL of water were subjected to the same treatment for 4 h. Next, the clay mineral suspension was mixed with the respective GO suspensions and sonicated for 4 h. The resulting suspensions were left under moderate magnetic stirring for 24 h. Finally, the remaining solids were separated from the gelatinous suspensions and washed with distilled water and ethanol by centrifugation for 30 min at 5000 rpm and were then subjected to drying in an oven at 60 °C for 24 h.

### Synthesis of the experimental RBCs

A total filler of 0.3% and 0.5% was incorporated into the experimental material for all groups, added slowly by manual mixing and homogenized for one minute. The materials were characterized by evaluating their 3-point flexural strength, modulus of elasticity, Fourier transform infrared spectroscopy (FTIR) and Knoop hardness.

The chemical composition of the experimental RBCs is presented in Table 1. A total of 7 groups were developed: G1 = control; G2 = resin matrix + 0.3% MMt (MMt/0.3); G3 = resin matrix + 0.5% MMt (MMt/0.5); G4 = resin matrix + 0.3% GO (GO/0.3);

 G5 = resin matrix + 0.5% GO (GO/0.5); G6 = resin matrix + 0.3% GO–MMt (GO–MMt/0.3) and G7 = resin matrix + 0.5% GO–MMt (GO–MMt/0.5).

### Fillers characterization

The filler morphology of GO functionalized with MMt (GO–MMt) was characterized and assessed using X-ray diffraction (XRD), FT–Raman spectroscopy, Fourier transform infrared spectroscopy (FTIR), transmission electron microscopy (TEM), and scanning electron microscopy (SEM) and was compared to GO and MMt alone.

#### X-ray diffraction (XRD)

The powder X-ray diffraction (XRD) patterns of the samples were recorded on a Shimadzu diffractometer 6000 model equipped with Cu*Kα* monochromatic radiation (λ = 0.15406 nm) operated at 40 kV and 30 mA. The XRD patterns were recorded over the 2θ range of 5°–80° with a step size of 0.02° s^-1^.

#### FT–Raman

FT–Raman spectra were obtained using a Renishaw brand Micro-Raman inVia spectrophotometer with an Ar + laser at a wavelength of 514 nm using a 50 × objective lens. Scanning was performed in the range of 400 to 4000 cm^-1^.

#### Fourier transform infrared spectroscopy (FTIR)

The FTIR spectra of the samples were recorded on an IR Prestige–21Shimadzu spectrophotometer in transmittance mode using the KBr pellet method. For each spectrum, a set of 30 consecutive scans were collected over the wavenumber range of 4000–400 cm^-1^ at a resolution of 4 cm^-1^. This analysis was conducted to verify the bands of the developed hybrid (GO–MMt). Such analysis was not performed in GO alone since the GO spectra are better observed through Raman analyses.

#### Transmission electron microscopy (TEM)

The morphology of MMt, GO and GO–MMt nanostructure powders was examined by TEM (FEI/Tecnai G^2^ F20, at 200 kV). For TEM evaluation, samples were dispersed in isopropyl alcohol and kept in an ultrasonic bath for 10 min for redispersion. The supernatant was dropped onto a support (copper grid with a C film), which was dried at room temperature for the analysis.

#### Scanning electron microscopy (SEM)

The nanomorphology of the MMt, GO and GO–MMt powders and samples measuring 2 × 2 mm^2^ prepared with mixtures of each material were monitored by SEM using an FEI Inspect S50 microscope at an accelerating voltage of 20 kV. The samples were fixed on carbon tape and coated with Au in a Quorum Model Q150R sputter-coater for 35 s at 20 mA by plasma generated under an argon atmosphere.

### RBC characterization

#### Contact angle

The samples were mounted separately on glass microscope slides using thin strips of Scotch®-Magic™ Tape (3 M) applied to the sample edges to obtain reasonably flat surfaces for measurement. Droplets of distilled water (MILIQ) (3 µl) were placed on the surface of each sample, and the contact angles were obtained by averaging the results of 3 measurements per sample, without repetitions in the same sample area. The wettability capacity was registered by a KSV Instruments, Ltd., equipment, model CAM101.

#### Flexural strength and modulus of elasticity

For the flexural strength and modulus of elasticity assessment, bar-shaped samples (8 × 2 × 2 mm^3^) (n = 6) were prepared by pouring the mixtures described above into stainless steel split-molds, according to ISO 4049 with modification of the sample length to avoid overexposure or uncured regions, considering the diameter of the LED curing device (1,000 mW/cm^2^ for 40 s; VALO; Ultradent, Utah, USA)^24^. The tests were conducted using a universal testing machine (Instron 5943, Norwood, MA, USA) equipped with a 500 N load cell using a crosshead speed of 0.5 mm/min. Values were determined with the following equation:

$$FS = 3FL/2bd^{2}$$

where FS is the flexural strength in MPa, *F* is the loading force at the fracture point, *l* is the length of the support span (6 mm), and *b* and *d* are the measured width and thickness, respectively.

#### Knoop microhardness determination

Disc-shaped samples measuring (4 × 2 mm^2^) (n = 6) were prepared by placing the material into stainless steel molds and covered with a polyester strip. The surface of the samples was polished using decreasing grit abrasive papers (in series 600, 800, 1200, Buehler Ltd., Lake Bluff, IL, USA) for 2 min each, followed by 0.5 mm diamond paste (Buehler Ltd., Lake Bluff, IL, USA). Three indentations were made on the top surface of each specimen along a middle line spaced 100 m$\mu$ from each other (Knoop diamond, 50 g, dwell-time 10 s) using digital microhardness equipment (Micromet II, Buehler, USA). The mean of the three readings was obtained for each sample.

#### Degree of conversion (DC)

The DC was evaluated by FTIR–ATR (FTIR 8400; Shimadzu) with a resolution of 4 cm^–1^ and 32 scans ranging from 4000^−1^ to 800 cm^−1^. Samples (n = 6) were analyzed after one minute of filler incorporation into the experimental materials. The mixture was immediately placed to cover the ATR crystal to obtain the baseline spectra. The material in the crystal was photoactivated for 40 s, and the spectral collection was repeated.

The percentage of unreacted carbon double bonds (C = C) was obtained from the peak height ratio of the methacrylate C = C (at 1638 cm^–1^) and those of an internal standard aromatic carbon double bond (at 1608 cm^–1^) during the polymerization in relation to the uncured material. The percentage of degree of conversion (% DC) was calculated for each sample as follows:

$${\text{DC }}\left( \% \right) = {1} - {\text{R3 min}}/{\text{Rbaseline}} \cdot {1}00$$

where R is the ratio of the absorbance at 1638 cm^−1^ to the absorbance at 1608 cm^−1^ as a function of time.

#### In vitro test in simulated body fluid (SBF)

This analysis was conducted in the hybrid modified RBCs. The samples (2 × 2 mm^2^) were inserted into a Teflon matrix and light-cured for 40 s {G1: resin monomers (negative control); G2 (monomers + 0.3% GO/MMt) and G3 (monomers + 0.5% GO/MMt)}. The solution used for the test was purchased from Sigma–Aldrich (phosphate buffered saline), pH 7.4. The pH of the solution was adjusted before the start of the test and during the period in which the blocks were immersed. The pH was measured and, if necessary, adjusted to 7.4. Three samples from each group of RBCs were immersed in the solution in an oven at 37 °C for 14 days, simulating the bioactivity test proposed by Kokubo and Takadama^25^. Until the EDX analysis, to verify whether there was deposition of Ca and P ions on the surface of the material, the samples were stored in a desiccator with silica. EDX analysis was conducted before immersion in solution and after 14 days of immersion.

### Statistical analysis

The data were statistically analyzed by using a statistical software program (Software R; R Core Team, 2020). The normality assumption of the residuals were verified using the QQ plot with a simulated envelope for flexural strength and microhardness analysis. Homoscedasticity assumption was verified from Predicted versus Residulas Plots. For DC and modulus of elasticity, Shapiro–Wilk tests were applied to evaluate the normality of the data. Flexural strength was analyzed using ANOVA, followed by Tukey’s HSD test. The Knoop hardness was analyzed by the Welch method for calculating the p value provided by ANOVA. The test of paired comparisons between groups was performed using the Sidak test for DC and sandwich-type estimators for modulus of elasticity. The level of significance was preset at $\alpha$= 0.05 for all quantitative analyses.

## Results

### Synthesis of hybrid GO–MMt composite

#### X-ray diffraction (XRD)

**Figure 1:**
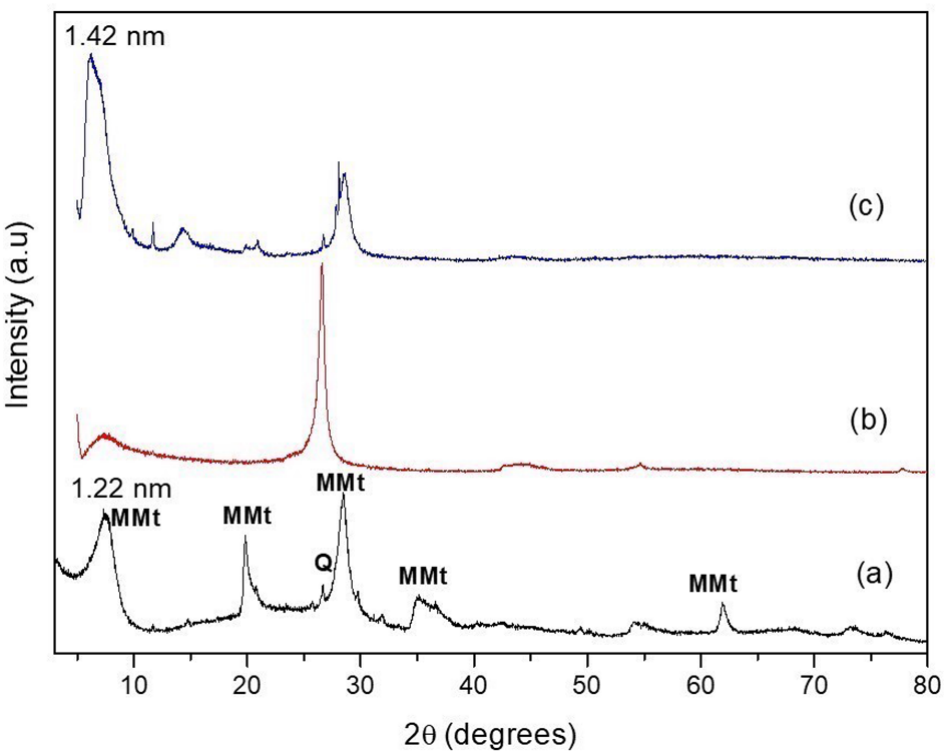
X-ray diffractograms: (**a**) MMt; (**b**) GO; and (**c**) GO–MMt.

Figure 1a shows the X-ray diffractogram of crude montmorillonite (MMt), showing a characteristic reflection plane of MMt and 2θ equal to 7.24°, which, according to the Bragg equation, is equivalent to a basal distance of 1.22 nm (d001)^26^. The GO X-ray diffractogram (Fig. 1b) showed an intense peak in the reflection plane (0 0 2), characteristic of graphite at 26.5° of 2θ, which is equivalent to a basal spacing of 0.34 nm, indicating that the material presents an ordered crystal structure and marked degree of reduction, characteristic of GO structures^27^. The GO–MMt hybrid (Fig. 1c), Fig. 1c, showed the presence of the characteristic peak of graphitic materials, which indicates the presence of GO in these materials. Peaks characteristic of clay minerals were observed, but with less intensity, indicating that the MMt crystal structure was not significantly affected, but an increase in basal spacing to 1.43 nm was observed, indicating possible intercalation processes of GO sheets between the clay mineral layers. The Q peak represents the quartz phase present in the MMt, as demonstrated in Fig. 1 by an intense reflection at 2θ equal to 25° and 27°.

#### Infrared spectroscopy (FTIR)

**Figure 2:**
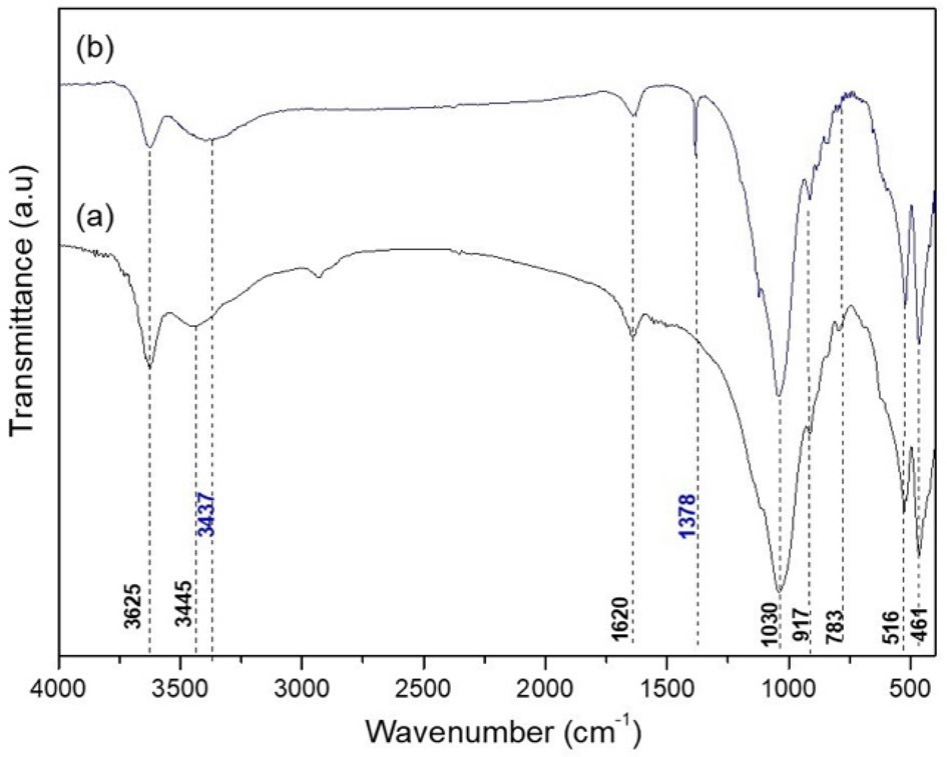
Infrared spectra: (**a**) MMt; and (**b**) GO–MMt.

Figure 2a shows the infrared spectra of MMt, highlighting an absorption at 3625 cm^-1^ referring to the stretching of structural OH groups of octahedral cations (M-OH, M = Al^3+^, Mg^2+^) and a broad band in the 3445 cm^-1^ region attributed to OH stretch vibrations of water and silanol groups (Si–OH)^28^. In the region of 1640 cm^-1^, a band referring to the angular deformation of water OH was observed. The presence of isomorphic substitutions can be evidenced by the presence of bands in the regions of 917 cm^-1^ and 783 cm^-1^, which are related to deformation vibrations of Al–Al–OH and Al–Mg-OH^29^. This analysis was conducted to verify the bands of the formed hybrid and clay. The FTIR in GO was not performed since the spectra would be better evaluated using Raman spectroscopy alone. In the spectrum of the developed hybrid GO–MMt, Fig. 2b, it was possible to observe that the bands referring to the inorganic structure of the clay mineral obtained small displacements, as observed in the band of 3445 cm^−1^, suffering moderate enlargement and displacement from its maximum to 3437 cm^−1^, which suggests a greater abundance of OH groups and may be an indication of possible interactions between OH groups from the GO and silanols from the clay mineral^23^. A new band at 1378 cm^-1^ indicates the presence of carboxyl groups (-CO-) from the GO structure.

#### Raman spectroscopy

**Figure 3:**
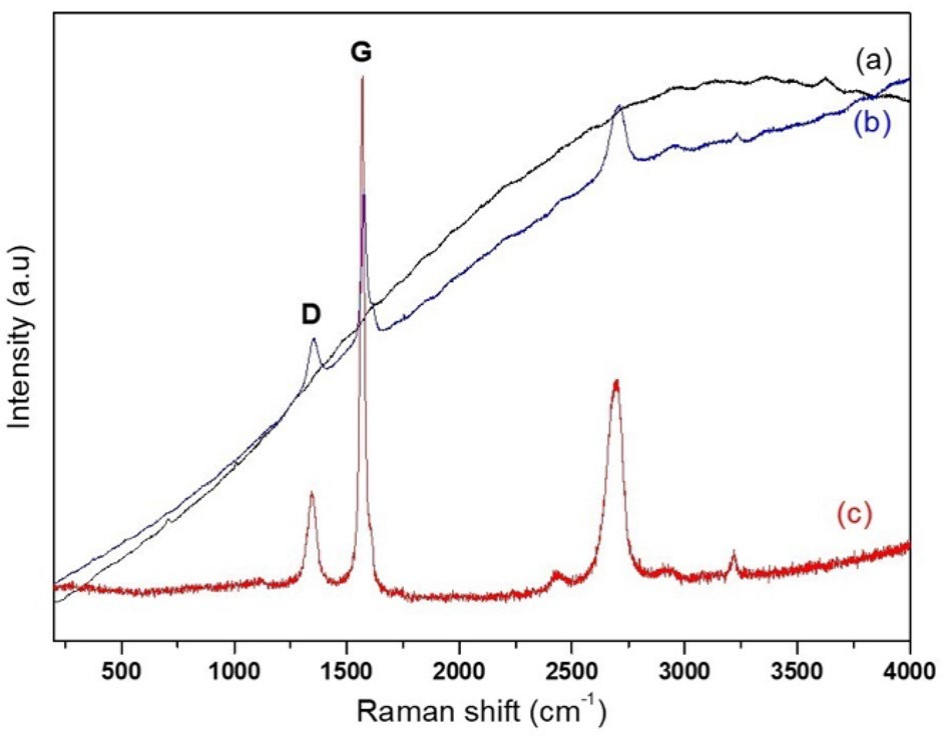
Raman spectra: (**a**) MMt; (**b**) GO; and (**c**) GO–MMt.

In Fig. 3, two characteristic signals of graphitic compounds are observed in the GO spectrum: bands at approximately 1568 and 1342 cm^-1^, which correspond to G bands, indicating a characteristic structure of graphite with sp2 carbon networks, and band D, which is characteristic of fault sites and sp^3^ networks.

#### SEM and EDS evaluation of hybrid GO/MMt nanoparticles

**Figure 4:**
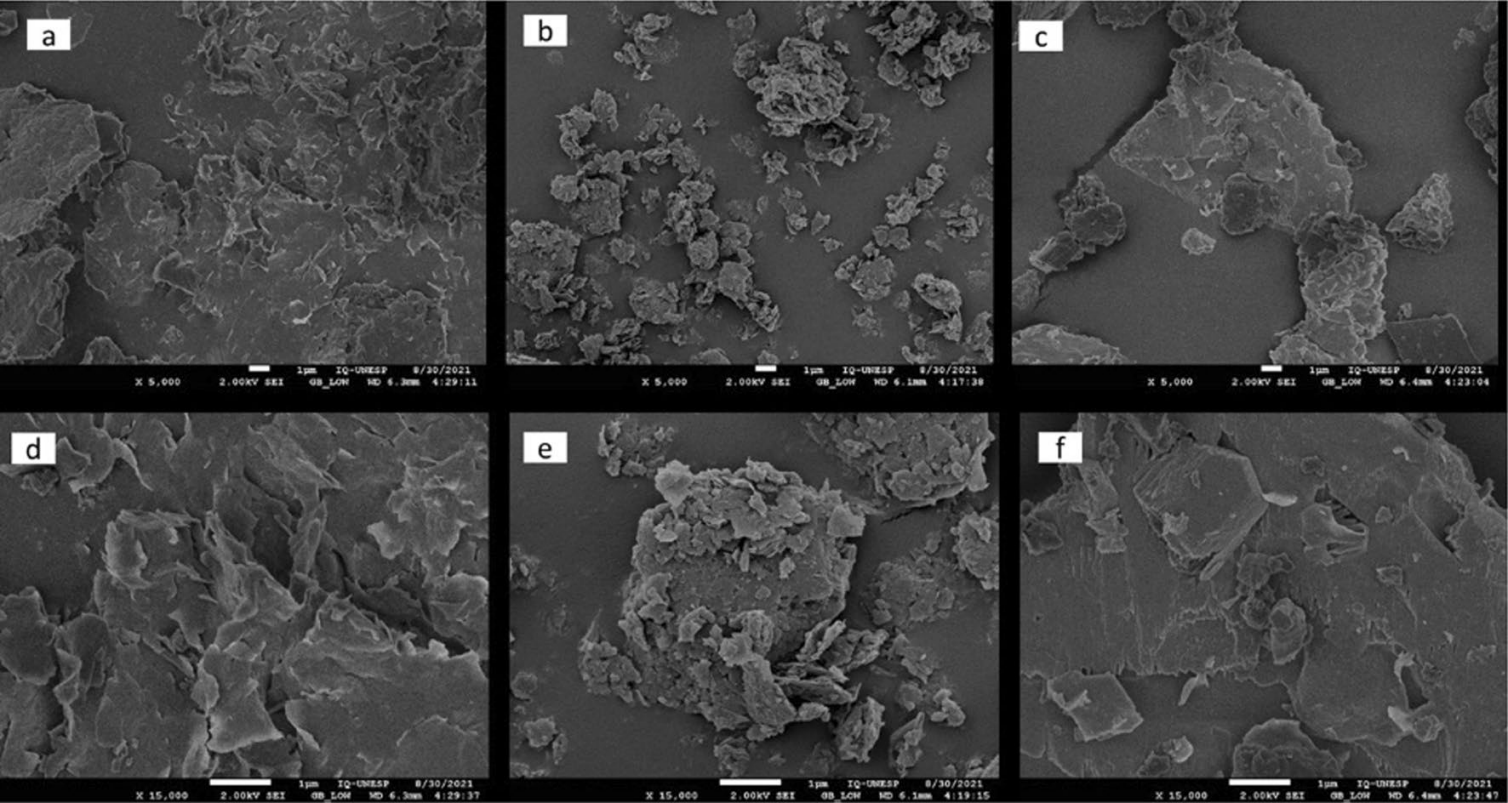
SEM images at 5000× magnification of (**a**) MMt nanoparticles, (**b**) graphene oxide (**c**) hybrid GO/MMt and 15,000×  magnification of (**d**) MMt nanoparticles, (**e**) graphene oxide, and (**f**) hybrid GO/MMt.

Figure 4 shows SEM images of the nanoparticles. The MMt surface (a, d) was observed to be more viscous, unlike the GO surface, which has overlaping nanosheets and particles (b, e). The hybrid images (c, f) show an intercalation of both phases on the surface.

**Figure 5:**
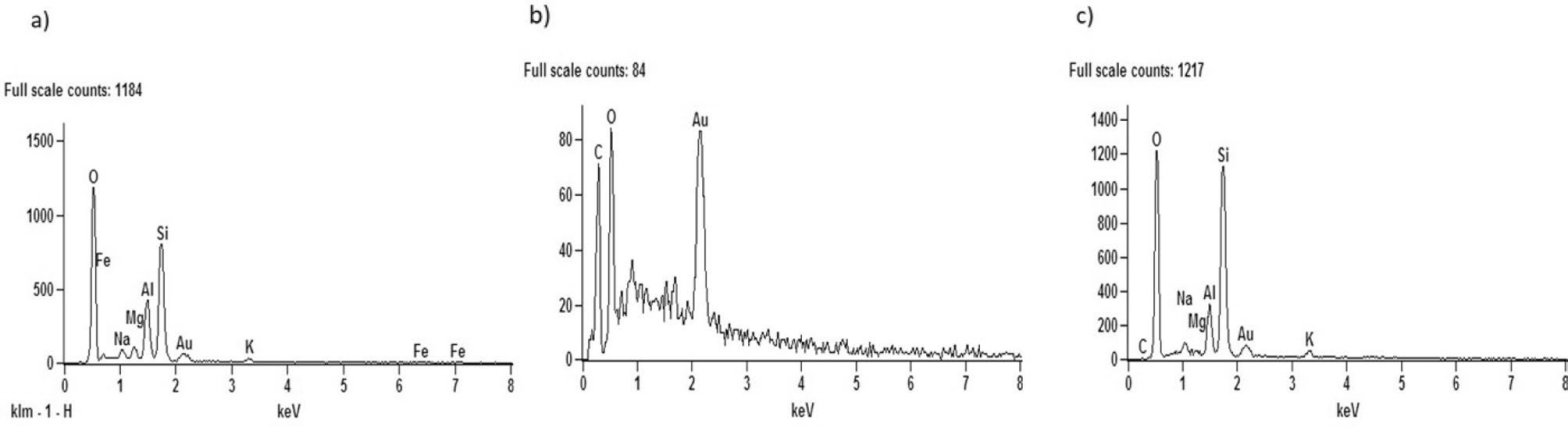
EDS analysis of the materials studied: (**a**) MMt, (**b**) GO, and (**c**) GO–MMt.

Figure 5 shows the EDS analysis of the nanoparticles. It is possible to observe the presence of magnesium (Mg), characteristic of clay, in the graph in Fig. 5a. Figure 5b shows the presence of oxygen (O) and carbon (C), constituents of graphene oxide. In the hybrid GO/MMt, the presence of all these components was observed (Fig. 5c). However, although the C peak was observed, it is a light structure to be verified by EDS, especially when there is an intercalation with another material, as in the case of the present study due to the presence of heavier materials, and therefore, the C is not highlighted the way it should be. Despite the limitation of the method for this assessment, it was possible to observe all components.

#### TEM evaluation

**Figure 6:**
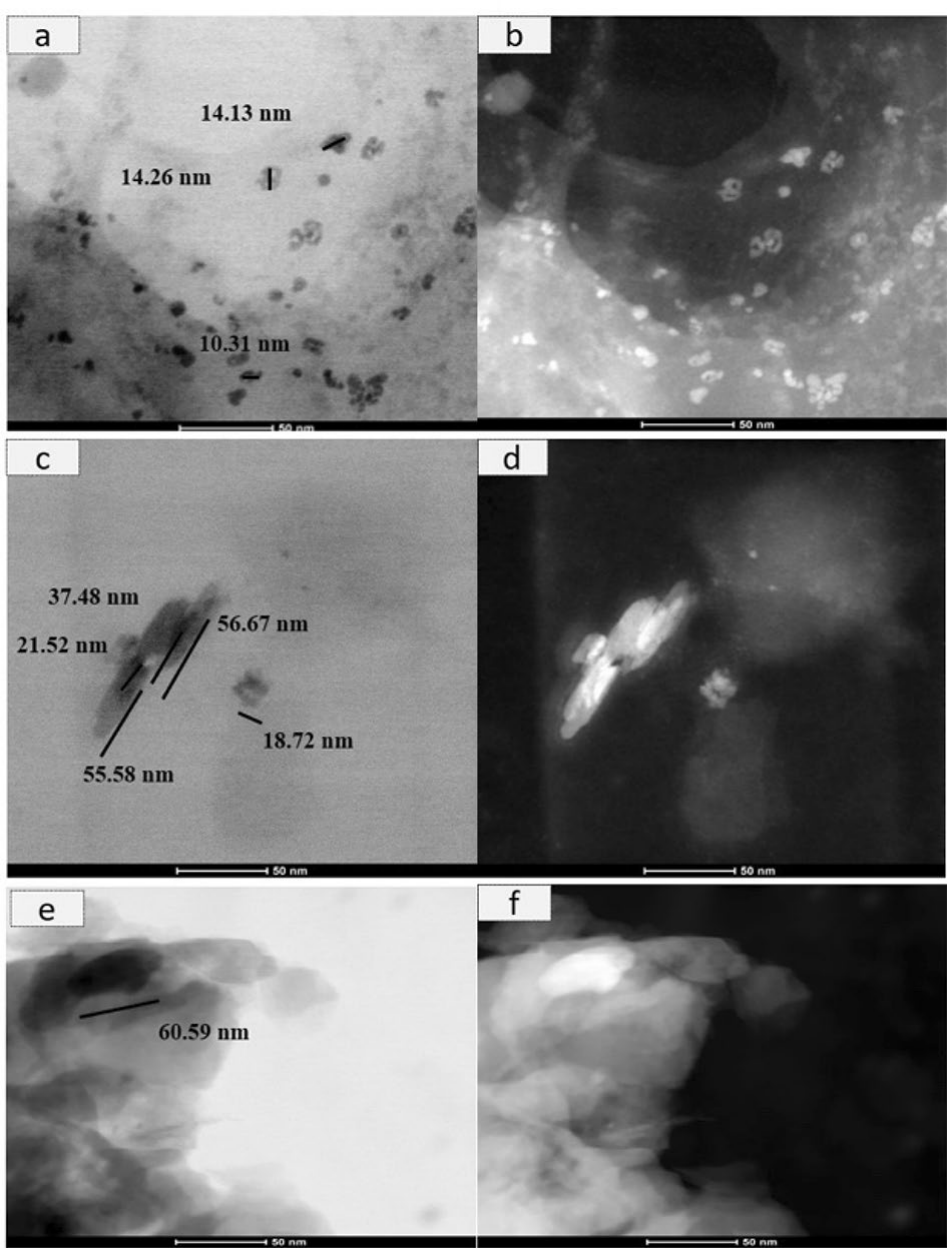
MET (magnification) of nanoparticles: brightfield (**a** (320,000×), **c** (450,000×), and (450,000×)) MMT, GO and hybrid GO/MMt and darkfield (**b** (320,000×), **d** (450,000×), **f** (450,000×)) MMT, GO and MMT/GO hybrid.

Figure 6 represents the morphology of the nanoparticles. Note that there was a varied mean size of nanoparticles in the overall surface dimensions evaluated. The areas of greater transparency indicate, in the image of the hybrid formed, the presence of a thicker nanoparticle due to the connection with the new functional groups, increasing the thickness of the hybrid. The hybrid shape is more similar to GO than MMt, with a more cylindrical shape, representing the deposition of GO nanosheets. In images c and d, which represent GO, aggregates of laminae of different thicknesses are observed, while MMt is presented in a spheroidal format.

**Figure 7:**
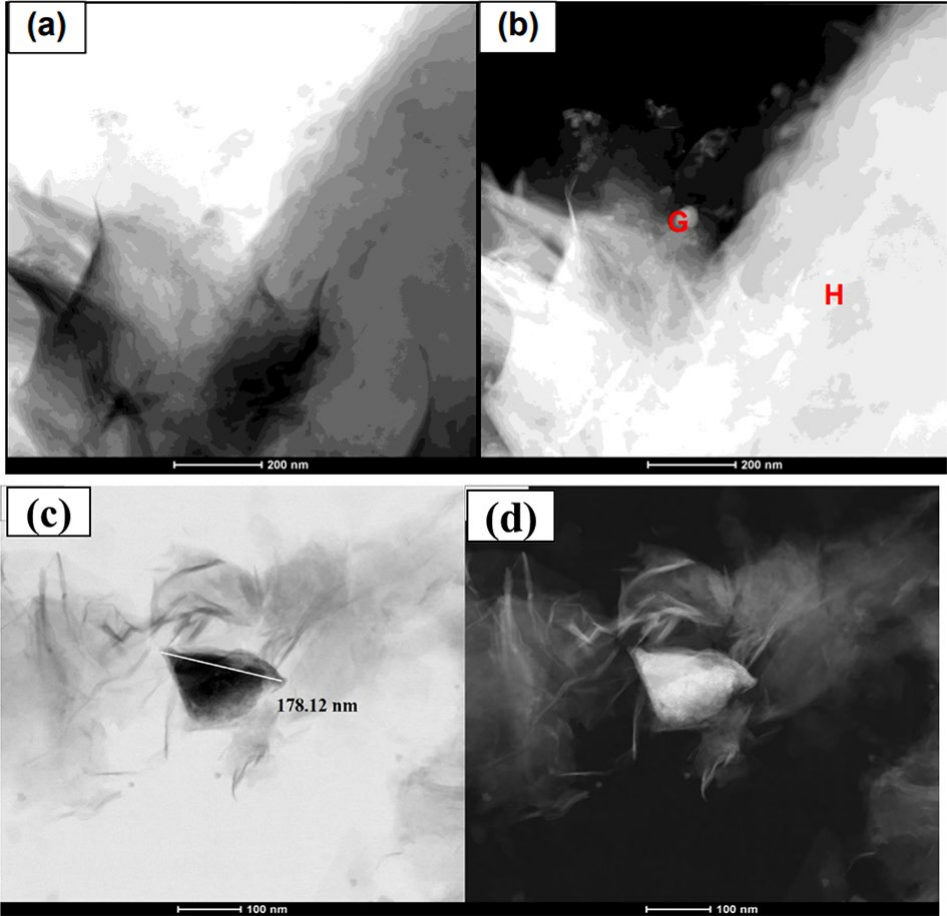
MET of GO/MMT hybrid nanoparticles: (**a**) brightfield (170,000×) and (**b**) darkfield (160,000×).

Figure 7 indicates the dispersion of the hybrid material in the GO nanosheets. Areas of medium transparency indicate overlapping of a greater number of layers^30^. The TEM micrographs in Fig. 7a, b show an interaction between MMt and GO, with a separation of the GO lamellar particles present in the hybrid. The points (G and H) demonstrate the morphology of the GO nanosheets and lamellar MMt nanoparticles. In Fig. 7c, it is also possible to observe a greater thickness of the hybrid of 178.12 nm, demonstrating the connection of the GO and MMt functional groups, which makes the nanoparticle thicker (Fig. 7c, d).

### Synthesis of the experimental RBCs

#### Contact angle, microhardness, flexural strength, modulus of elasticity and degree of conversion

Table 2 reflects the results regarding the contact angle, microhardness, flexural strength, modulus of elasticity and degree of conversion of the experimental RBCs.

**Table 2:** Contact angle (°), microhardness (kg/mm^2^), flexural strength (MPa), modulus of elasticity (GPa) and degree of conversion (%) values (mean ± SD) for the RBCs.

Groups	Contact angle (n = 3)	Microhardness (n = 6)	Flexural strength (n = 6)	Modulus of elasticity (n = 6)	Degree of conversion (n = 6)
G1	91.24 ± 8.50	35.2 ± 1.8^a^	69.8 ± 1.98^bc^	3.49 ± 0.21^f^	61.9 ± 0.20^ab^
G2	84.28 ± 3.33	47.9 ± 2.1^b^	65.7 ± 4.36^bc^	0.78 ± 1.16^a^	58.9 ± 2.45^a^
G3	77.12 ± 3.29	52.0 ± 2.8^bc^	77.5 ± 1.93^c^	3.12 ± 1.42^f^	66.0 ± 0.19^b^
G4	84.17 ± 4.39	54.8 ± 3.0^c^	60.6 ± 2.90^b^	2.64 ± 0.21^c^	62.1 ± 1.18^ab^
G5	85.97 ± 3.28	49.1 ± 4.6^bc^	47.00 ± 2.00^a^	1.14 ± 0.29^b^	62.8 ± 3.46^ab^
G6	85.07 ± 7.57	65.1 ± 1.8^d^	100.1 ± 3.52^e^	2.84 ± 0.91^e^	66.2 ± 0.20^b^
G7	76.11 ± 4.58	68.6 ± 1.6^e^	86.1 ± 0.93^d^	2.99 ± 0.10^d^	59.5 ± 1.12^a^

Values in the same column with different superscript lower − case letters significantly differ from each other (*p* < 0.05).

Regarding flexural strength data, G6 showed superior results, followed by G7 (Table 2, *p* < 0.05). It is also observed that the GO at a concentration greater than 0.5% (G5) showed a difference from the control, with lower flexural strength values, indicating that the passage of light in this darkened particle was difficult, decreasing the mechanical properties. This did not happen with the developed hybrid since it had a light coloration (G6 and G7 groups).

Table 2 also shows the results regarding the material's contact angle, hardness, modulus of elasticity and degree of conversion. We can observe that all study groups had a contact angle of less than 90°, characterizing hydrophilic materials, with the exception of the control group, which was hydrophobic. Such results are favorable since for the biopolymer to present bioactivity, it is necessary that it releases the ions from the organic matrix with the medium, and therefore, the more soluble the material is, the more bioactive potential it presents.

Regarding the surface hardness, the control group (G1) had lower hardness than all other groups (*p* < 0.0001). The microhardness observed for the G7 group was higher than that observed for all other groups (*p* = 0.0182 for the comparison with G6 and *p* < 0.0001 for all other comparisons). The G6 group also presented higher microhardness than the clay, control and graphene groups (*p* < 0.0001).

For the modulus of elasticity, the G2 group composed of clay did not show more rigidity, which was expected since clay does not positively influence the mechanical properties of the material. The G4 and G5 groups formed by GO showed a lower modulus of elasticity.

#### SEM analysis

**Figure 8:**
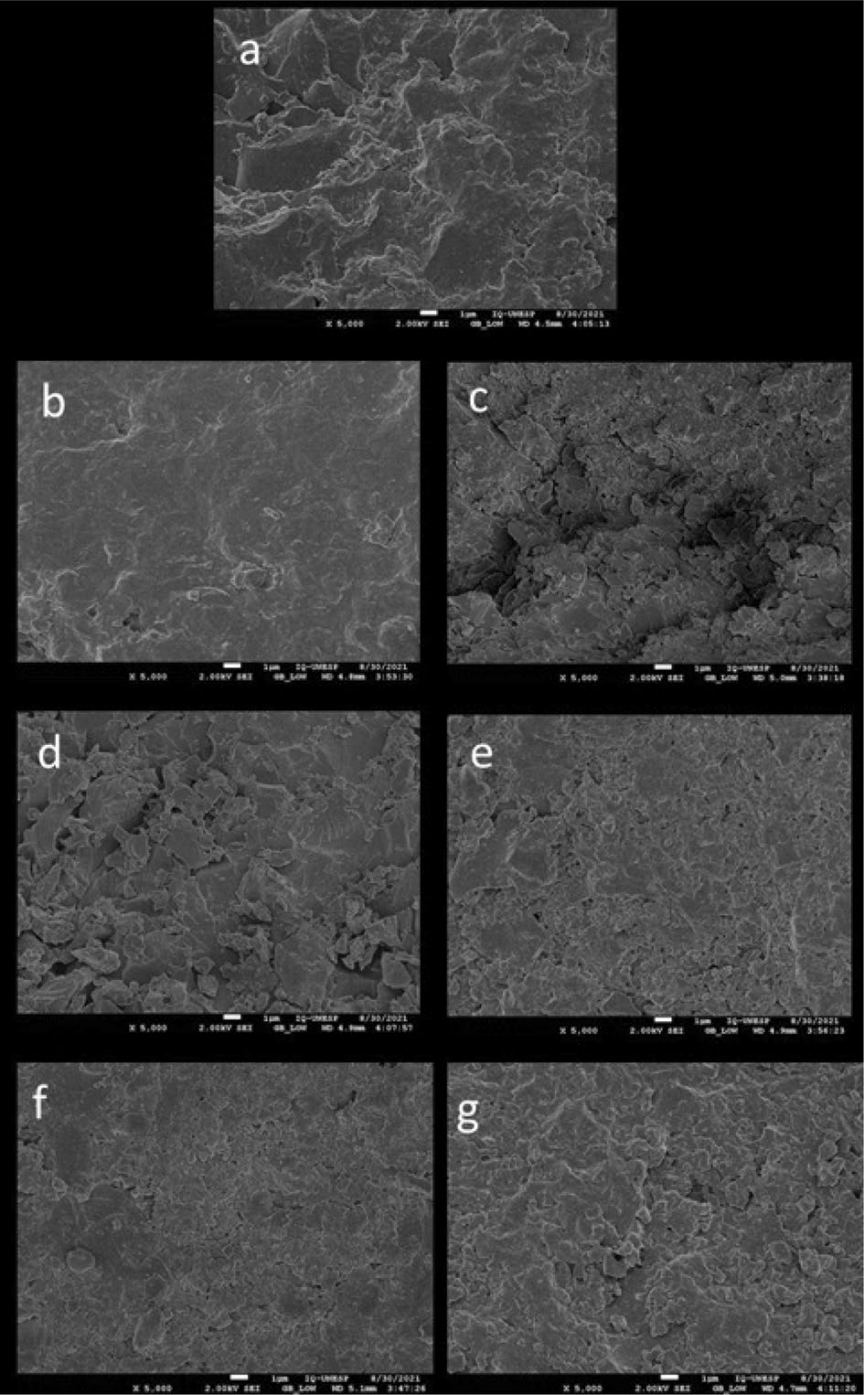
SEM images (5000×) of RBCs synthesized with nanoparticles: (**a**) G1: control; (**b**) G2: resin matrix + 0.3% MMt (MMt/0.3); (**c**) G3: resin matrix + 0.5% MMt (MMt/0.5); (**d**) G4: resin matrix + 0.3% GO (GO/0.3); (**e**) G5: resin matrix + 0.5% GO (GO/0.5); (**f**) G6: resin matrix + 0.3% GO–MMt (GO–MMt/0.3) and (**g**) G7: resin matrix + 0.5% GO–MMt (GO–MMt/0.5).

Figure 8 shows the SEM images of the RBCs with nanoparticles after the longitudinal section of the resin specimens. It is possible to observe that on the surface with 0.5% MMt (Fig. 8c), there are voids and cracks when compared to the control (Fig. 8a) or MMt0.3 (Fig. 8b). In the GO images (Fig. 8d–e), it is possible to observe a better distribution of particles, while on the surface of the hybrid GO/MMt 0.3% (Fig. 8f) homogeneity is observed, which characterizes a better dispersion and interaction with the organic matrix, even when compared to the RBC with 0.5% GO/MMt (Fig. 8g).

#### In vitro test in simulated body fluid

**Figure 9:**
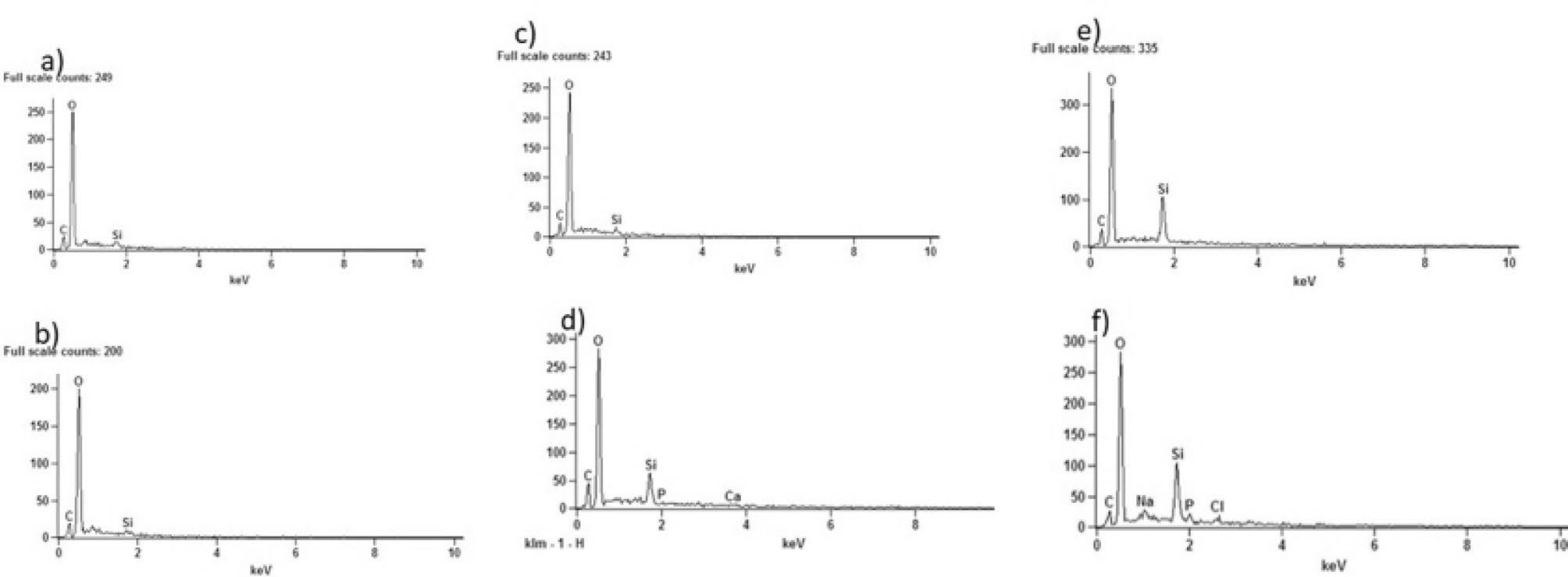
Evaluation of calcium (Ca) and phosphate (P) ion deposition by EDX in (**a**) G1 before immersion in simulated body fluid (SBF); (**b**) G1 after 14 days of immersion in SBF; (**c**) G2 before immersion in SBF; (**d**) G2 after 14 days of immersion in SBF; (**e**) G3 before immersion in SBF; and (**f**) G3 after 14 days of immersion in SBF.

**Figure 10:**
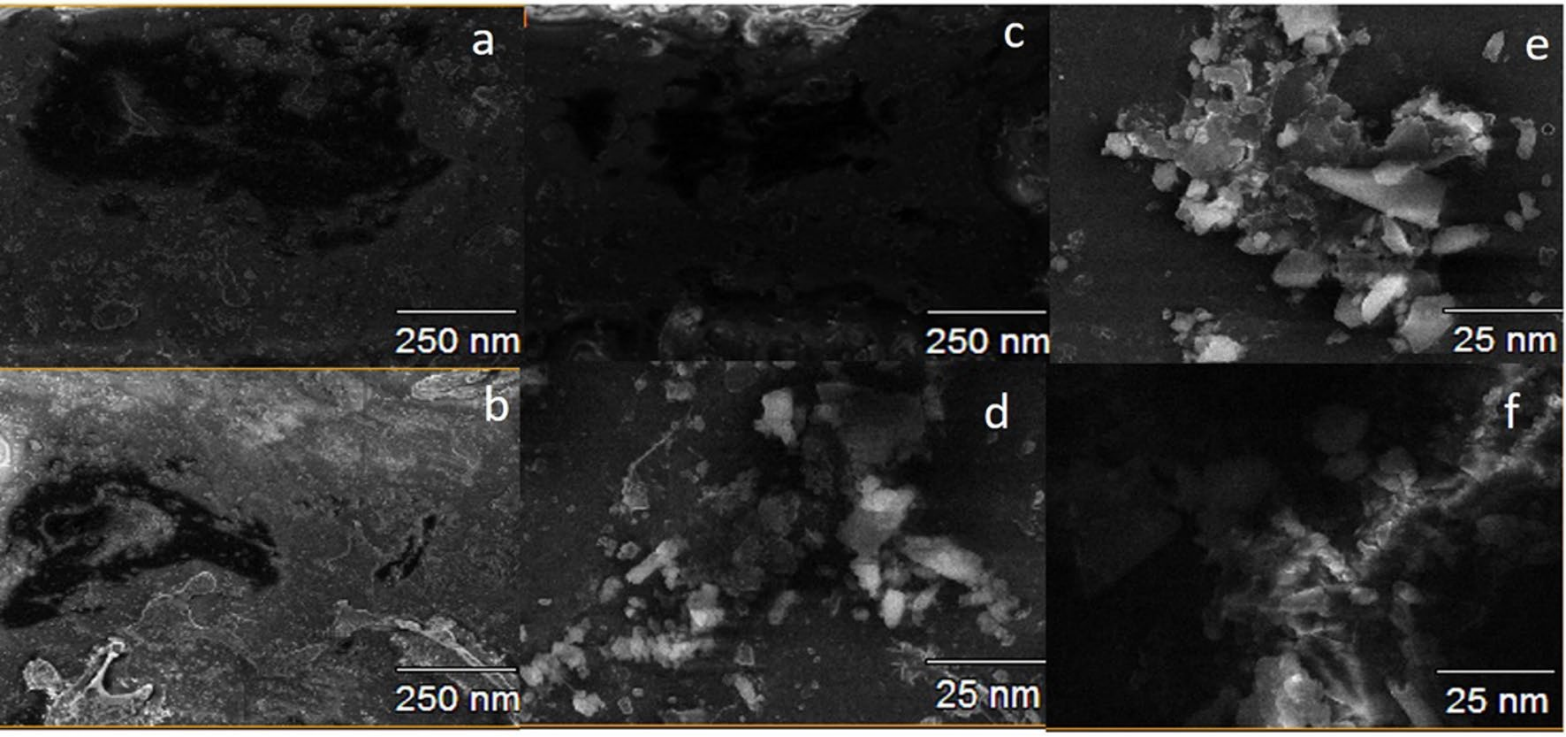
SEM (10,000×) analysis of biopolymer morphology in the bioactivity test: (**a**) G1 before immersion in simulated body fluid (SBF); (**b**) G1 after 14 days of immersion in SBF; (**c**) G2 before immersion in SBF; (**d**) G2 after 14 days of immersion in SBF; (**e**) G3 before immersion in SBF; and (**f**) G3 after 14 days of immersion in SBF.

Figure 9a, c and e reflect the groups with the hybrid before immersion in the solution, while Fig. 9b, d and f represent the groups after 14 days of immersion in the solution. Figure 9d and f show that the groups formed by the hybrid showed calcium (Ca) and phosphate (P) deposition on the surface, different from the control after 14 days of immersion in the solution (Fig. 9b). It is possible to say that there is a bioactive potential in this material that deserves further investigation. In addition, in Fig. 10, it is possible to observe the presence of particles on the surface in the monomers with the hybrid after 14 days (d, f), suggesting calcium phosphates.

## Discussion

The development of hybrid nanomaterials has been highlighted in recent decades for integrating the properties of different constituents to obtain optimized characteristics, including electronic, chemical, optical, thermal and mechanical properties. In general, graphene and its derivatives can be blended with polymers via several routes to develop a composite with improved properties and achieve good dispersion^31^. Previously, Ioannidis et al.^10^ evaluated the antimicrobial efficacy of silver nanoparticles (AgNPs) synthesized on an aqueous GO matrix against endodontic biofilms, presenting improved microbial killing efficacy, as a potential biomaterial for root canal irrigation. The synthesis of nanofillers of zirconia functionalized with GO was also successfully produced as a promising nanoparticle for improving the performance of restorative systems^12^, which boosted new research using hybrid composites with GO in the dental materials field.

Although graphene and GO have been shown to significantly improve the physicochemical and biological properties and antimicrobial attributes of biomaterials^10,32,33^, graphene sheets are not homogeneously dispersed into the matrix, which limits their application as mechanical reinforcements in composite systems. This agglomeration occurs due to graphene's strong hydrophobicity and van der Waals attraction^34^, which aggregates graphene sheets into flakes of weakly interacting monolayered sheets, affecting the enhancement in the matrix^8^. In this way, ultrasound syntheses have been widely adopted^23^ for the development of hybrid GO materials, consisting of compression and rarefaction cycles in which the negative acoustic pressure can be high enough to disrupt liquid interactions, providing energy to separate the graphite layers^35^. Since MMt is known for its adsorption and intercalation capacity, the properties of these materials allow interesting routes for the synthesis of hybrids with GO, enabling the insertion of carbon precursors between silicate layers^35^.

In the current study, we successfully synthetized a hybrid composite of GO–MMt via ultrasound treatment and direct contact with aqueous suspensions to promote a suspension with good dispersity and provide stability to the carbonaceous layer structure of graphene, in addition to improving the physicochemical properties, extending the application of GO in dental materials. The hybrid obtained (GO–MMt) (Fig. 1) presented the characteristic peaks of clay minerals, but with less intensity, suggesting an increase in the basal spacing to 1.42 nm, indicating that GO layers suffered intercalation between the layers of clay minerals. Another factor that shows the intercalation of both materials is the absence of the characteristic GO peak in the hybrid diffractogram. The FTIR analysis of the developed material, GO–MMt (Fig. 2), showed bands referring to the inorganic structure of the clay mineral with small displacements and moderate enlargement in the hybrid spectrum. Sustaining the intercalation process denotes interaction processes between the inorganic matrix and the carbonaceous material, such as electrostatic interactions between GO and the basal oxygen surface of MMt, as well as with interlamellar cations^36^. The enlargement of the characteristic peak of MMt is indicative of a disorganization of the clay mineral structure, which may be a consequence of possible interactions between GO with the outer surface and edge region of MMt, which is an interesting reactivity region in this silicate^23,36,37^. TEM micrographs (Fig. 7a, b) also show an interaction between MMt and GO, with a separation of the GO lamellar particles present in the hybrid. At points (G and H), the morphology of the GO nanosheets and lamellar MMt nanoparticles is observed. The characterizations presented herein suggested that the hybrid material was synthetized*,* although this is the first exploratory and preliminary study aiming to understand a series of events related to the synthesis of the hybrid GO/MMt and the development of a dental resin-based composite modified with this hybrid material.

Raman spectroscopy has been widely used in the characterization of graphene compounds^38^. The presence of two bands (G and D) is observed in Fig. 3. The D-band results from the oxidation process, which modifies the structure of graphite by the formation of several functional groups, generating several defects, such as extension of bonds, changes in the order of edges, cavities and bonding disorder, and is used for characterization of GO^37^. The relationships between the G and D bands can be used to assess the degree of oxidation of the GO layers.

In the GO spectrum (Fig. 3), the G band presents a relatively higher intensity, which indicates a greater extension of Csp2 graphitic structures, which are characteristic of GO structures, and corroborates the XRD analysis (Fig. 1). The clay mineral MMt usually does not show characteristic signs in Raman spectroscopy, which can be evidenced in the presented spectrum. On the other hand, the hybrid GO–MMt material maintains the characteristic GO bands, verified with less intensity, which indicates the presence of the carbonaceous material in the hybrid obtained. Such results suggest that graphene oxide was intercalated with MMt, showing a more stable compound and offering one reliable experimental material.

EDS analyses showed the presence of both MMt and GO components (Fig. 5c). However, although the carbon (C) peak was observed, since it is a light structure, it is not fully detected by EDS, especially when there is an intercalation with another material, as in the case of the present study, due to the presence of more heavy materials. Thus, C was not highly highlighted in the hybrid by EDS. Despite the limitation of the method for this assessment, it was possible to observe all components. Regarding the size of the nanoparticles evaluated by MET, the larger size of the hybrid (Fig. 6e, f) and (Fig. 7a, b) compared to the MMt and GO nanoparticles alone indicates that there was binding of the GO and MMt functional groups, which makes the nanoparticle thicker, and the analyses conducted allowed verification that the GO–MMt hybrid was successfully developed to be explored.

Another interesting feature of the GO–MMt hybrid is its coloration, featuring a highly light brown color. The literature shows that graphene and its derivatives are highly dark and promote a change in the color of polymers, even at low concentrations (less than 0.1%), which limits their application in the development of biopolymers^2,31^. Therefore, some studies have limited the amount of GO in resin-based composites due to limitations related to aesthetics^39,40^. Owing to the improvements in the mechanical properties related in the present study and considering the color of the hybrid, a new set of applications could be started using the GO–MMt hybrid in dental materials, and this material will be suitable for further research for aesthetic purposes.

In the present study, nanoparticle concentrations of 0.3% and 0.5% were used for the synthesis of the experimental resins. It was demonstrated that the fraction of the bioactive fillers in the RBCs should be kept to a minimum to promote remineralization through ion release without affecting their mechanical properties^41,42^. Although the concentrations used were low, it has been previously shown that the addition of just 0.1–5% graphene by weight to RBCs is sufficient to increase their mechanical properties^43^. The low concentrations of nanoparticles used in the present study made it difficult to visualize the sectioned specimens by SEM (Fig. 8). However, it was possible to observe that since there was an increase in concentration, there was a greater appearance of voids between the organic matrix of the monomers, which could negatively influence the mechanical properties. It is also important to note that obtaining high strength characteristics after adding nanoparticles depends not only on the absence of agglomerates but also on high adhesion of the filler and chemical interactions with the matrix. In relation to the chemical interaction with the matrix, Fig. 5 (EDS analysis of the materials) indicates the presence of Si (Fig. 5c—GO/MMt); since MMt is an aluminosilicate mineral and because of its hydrophilicity, it has positive effects on adsorption performance^21^. These results also explain the higher flexural strength values demonstrated by G6 compared to G7 (*p* < 0.05; Table 2) because if nanoparticles were not dispersed adequately, the mechanical properties of the resin with GO/MMt were not increased, as shown by our results, especially flexural strength, which immediately decreased if there was some agglomeration or low distribution of the nanoparticles^24^. In this study, flexural strength was determined as the main indicator of the physical–mechanical properties of RBCs, since it develops tensile, compressive and shear stresses during the test, and testing the material under its most challenging mechanical situation may lead to a reduced chance of accepting a material that fails prematurely due to inadequate strength^44^.

G5, which was composed of GO at higher concentrations (0.5%), had lower flexural strength values than the control group (Table 2). During the preparation of the experimental RBC in this group (G5), a greater agglomeration of particles was noticed, and by presenting higher concentrations of GO (0.5%), a highly dark black color was observed. This indicates that the passage of light during photoactivation of monomers with higher concentrations of darkened particles was hampered, decreasing the mechanical properties, and that there was probably an agglomeration of GO particles at higher concentrations. The same did not occur in the RBCs formed by the hybrid GO–MMt material, either at a concentration of 0.3% or 0.5%, as shown in Table 2, which demonstrates that the GO was stabilized in the presence of MMt, dispersing better in the resin matrix. In addition, due to the lighter color of the material developed, the passage of light during the photoactivation procedure was facilitated, interacting better with resin monomers.

Although the G6 group showed higher flexural strength values than G7, the G7 group demonstrated a higher surface microhardness value than G6 (*p* = 0.0182, Table 2). This confirms that even at higher concentrations (0.5%), the incorporation of the hybrid into the RBC occurred, probably without agglomeration of the nanoparticles. Such results were confirmed with the images of the surface of the hybrid GO/MMt 0.3% and 0.5% (Fig. 8f, g), in which there is homogeneity, which characterizes a better dispersion and interaction with the organic matrix.

Contrary to these results, the G6 and G7 groups showed lower modulus of elasticity values than the control group (G1), with a significant difference (*p* < 0.05, Table 2). As the strongest material known to date, graphene has an elastic modulus of 1 TPa^45,46^. A limitation of the present study is that functionalized graphene was not used, and despite stabilization with MMt for hybrid development and all the advantages of the hybrid already discussed above, there may have been a level of agglomeration that impaired the evaluation and performance of this material in terms of modulus of elasticity. On the other hand, the low nanoparticle concentrations used may have contributed to the elastic modulus results. As in the present study, which found values of G6 (0.3% GO–MMt) (2.84 ± 0.91 GPa) and G7 (0.5% GO–MMt) (2.99 ± 0.10 GPa) for the modulus of elasticity (Table 2), the research previously performed by Ruan et al.^47^ also found a value of 2.8 GPa when only 0.2% GO was added to carboxymethyl–chitosan^47^. Another study^48^ demonstrated that a high concentration of GO sheets (6%) increased the modulus of elasticity of the material from 2.4 to 6.3 GPa, supporting the hypothesis that high concentrations of GO nanoparticles or GO, as in the present study, are necessary for an increase in the modulus of elasticity of the material to occur.

The statistical analysis did not show a significant difference between G6 or G7 in relation to the control (*p* > 0.05) in the DC analyses, but an increase was observed for the groups formed by the hybrid with 0.3% (G6), as well as the G3 group (MMt/0.5%). Such an increase may be related to the photocatalytic characteristics of GO, increasing the system's reactivity and polymerization rate^49^. In the present study, the groups with GO (G4 and G5) did not present significant differences from the other groups. These properties are essential to ensure the mechanical resistance of a biomaterial, and therefore, the DC was evaluated in the present study. We hypothesized that the values could have a significant difference if evaluated over a longer period of time after incorporation of the nanoparticles and not immediately after, allowing a better dispersion and reaction with the monomers, but more studies are needed to confirm this hypothesis.

Regarding the wettability, although graphene is known to be a hydrophobic material^50^, the analysis of RBCs formed by GO or by the hybrid GO–MMt were characterized in the present study as hydrophilic materials, in which all study groups obtained a contact angle less than 90°, with the exception of the control group, which presented a value of 91.24 ± 8.50 (Table 2), characterizing a hydrophobic resin matrix. For contact angles less than 90°, the material is considered hydrophilic, while angles greater than 90° are hydrophobic or water repellent^51^. When the material becomes more hydrophilic, the biopolymer can present bioactivity since it releases the ions from the organic matrix into the medium, and therefore, the more soluble the material is, the more bioactive potential it presents^52^.

Considering the promising results shown herein regarding the mechanical properties of RBCs composed of the hybrid of GO–MMt, we also evaluated their bioactive potential. To simulate biomimetic mineralization, simulated body fluid (SBF) was used to provide suitable temperature, ion concentration, and pH conditions similar to those of human blood plasma. SBF offers a suitable supersaturated environment around the substrates and facilitates the deposition of bone-like apatite [54]. Although EDX analysis cannot evaluate the functional groups formed on the surface of the material, it was possible to detect different elements. The RBCs formed by GO–MMt showed deposition of Ca and P ions on the surface (Fig. 9d, f) after 14 days of immersion in SBF. Thus, a bioactive potential in this material was found and deserves further investigation through the evaluation of functional groups and the formation of hydroxyapatite. The presence of particles on the surface suggesting calcium phosphates (Fig. 10) was also observed in the SEM images.

A limitation of the SBF bioactivity test in the present study is that the RBC specimens were lightly polished. A factor that contributes to the deposition of apatite on the surface is the increase in roughness, since a rough surface has a large surface area, promoting greater contact with the SBF solution, the apatite deposition process accelerates^52^. Studies related to material sorption and solubility should also be conducted since greater solubility is needed in bioactive materials, although the clinical performance of the material depends on a low solubility^52^. It will be of great interest to compare these results with commercial RBCs in future research. Our main objective herein was to evaluate experimental resin monomers without filling them, as experimental monomers allowed us to evaluate different components of the material under controlled conditions to evaluate the effect of the hybrid as reinforcement. Despite the difficulty of comparison with commercial composites because of the variability in composition, the current results can be used as standards and can be addressed by developing experimental resin composites with a mixture of several monomers that resemble commercial formulations. Thus, further studies verifying the performance of these RBCs will be conducted since the addition of the GO–MMt hybrid in RBCs, with improvements in the mechanical, aesthetic and bioactive properties, opens up a range of future applications in the field of dental materials.

## Conclusion

The hybrid obtained (GO–MMt) was successfully incorporated into a resin-based composite (organic matrix). The resin composed of 0.3% GO/MMt presented superior flexural strength results compared to the 0.5% GO/MMt, a homogeneous dispersion in the surface and higher microhardness values compared to the clay, control and graphene materials. The resin-based composites filled by GO–MMt had a contact angle of less than 90°, were considered hydrophilic materials and showed deposition of Ca and P ions. Overall, resin-based composites composed of the hybrid showed promising results in terms of mechanical properties and bioactive potential, extending the application of GO in dental materials.

## Supplementary Information

Supplementary Information 1.

Supplementary Information 2.

Supplementary Information 3.

Supplementary Information 4.

Supplementary Information 5.

Supplementary Information 6.

Supplementary Information 7.

Supplementary Information 8.

Supplementary Information 9.

Supplementary Information 10.

Supplementary Information 11.

Supplementary Information 12.

## Data Availability

All data is given in the supplementary file.
