# Supplementary material for: Enhancing the mechanical properties and providing bioactive potential for graphene oxide/montmorillonite hybrid dental resin composites
Source: Sci Rep. 2022 Jun 17;12:10259. doi: 10.1038/s41598-022-13766-1 (PMC9205868; doi:10.1038/s41598-022-13766-1)
Supplement: Supplementary file 1 — Supplementary Information 1. [file 41598_2022_13766_MOESM1_ESM.pdf]

| **GRUPOS** | Grau de conversão |
|---|---|
| Controle | 62,02 |
| Controle | 61,74 |
| Controle | 61,9 |
| Controle | 62,8 |
| Controle | 61,4 |
| Controle | 61,63 |
| Argila 0.3 | 57,35 |
| Argila 0.3 | 62,24 |
| Argila 0.3 | 55,59 |
| Argila 0.3 | 58,76 |
| Argila 0.3 | 59,78 |
| Argila 0.3 | 59,68 |
| Argila 0.5 | 60,65 |
| Argila 0.5 | 70,57 |
| Argila 0.5 | 72,33 |
| Argila 0.5 | 71,76 |
| Argila 0.5 | 60,46 |
| Argila 0.5 | 60,28 |
| Grafeno 0.3 | 60,32 |
| Grafeno 0.3 | 60,91 |
| Grafeno 0.3 | 62,6 |
| Grafeno 0.3 | 67,6 |
| Grafeno 0.3 | 61,84 |
| Grafeno 0.3 | 59,51 |
| Grafeno 0.5 | 65,78 |
| Grafeno 0.5 | 59,67 |
| Grafeno 0.5 | 64,51 |
| Grafeno 0.5 | 64,84 |
| Grafeno 0.5 | 63,43 |
| Grafeno 0.5 | 58,27 |
| Hibrido 0.3 | 65,73 |
| Hibrido 0.3 | 65,22 |
| Hibrido 0.3 | 65,27 |
| Hibrido 0.3 | 66,89 |
| Hibrido 0.3 | 67,27 |
| Hibrido 0.3 | 67 |
| Hibrido 0.5 | 59,69 |
| Hibrido 0.5 | 52,88 |
| Hibrido 0.5 | 52,78 |
| Hibrido 0.5 | 63,77 |
| Hibrido 0.5 | 62,81 |
| Hibrido 0.5 | 65,05 |
