# Supplementary material for: Enhancing the mechanical properties and providing bioactive potential for graphene oxide/montmorillonite hybrid dental resin composites
Source: Sci Rep. 2022 Jun 17;12:10259. doi: 10.1038/s41598-022-13766-1 (PMC9205868; doi:10.1038/s41598-022-13766-1)
Supplement: Supplementary file 5 — Supplementary Information 5. [file 41598_2022_13766_MOESM5_ESM.pdf]

AMOSTRAS	VALORES
CONTROLE	62,14
CONTROLE	65,61
CONTROLE	77,41
CONTROLE	70,13
CONTROLE	71,48
CONTROLE	71,92
ARGILA 0.3	51,25
ARGILA 0.3	55,6
ARGILA 0.3	68,6
ARGILA 0.3	60,97
ARGILA 0.3	79,01
ARGILA 0.3	78,6
ARGILA 0.5	70,08
ARGILA 0.5	74,78
ARGILA 0.5	74,33
ARGILA 0.5	81,9
ARGILA 0.5	81,46
ARGILA 0.5	82,68
GRAFENO 0.3	62,27
GRAFENO 0.3	68,99
GRAFENO 0.3	64,45
GRAFENO 0.3	65,97
GRAFENO 0.3	51,57
GRAFENO 0.3	50,36
GRAFENO 0.5	39,87
GRAFENO 0.5	53,64
GRAFENO 0.5	46,36
GRAFENO 0.5	52,3
GRAFENO 0.5	42,48
GRAFENO 0.5	47,65
M1R 0.3	117,21
M1R 0.3	102,14
M1R 0.3	101,7
M1R 0.3	93,55
M1R 0.3	93,6
M1R 0.3	92,14
M1R 0.5	85,46
M1R 0.5	84,02
M1R 0.5	89,4
M1R 0.5	88,46
M1R 0.5	82,96
M1R 0.5	86,48
