# Supplementary material for: Enhancing the mechanical properties and providing bioactive potential for graphene oxide/montmorillonite hybrid dental resin composites
Source: Sci Rep. 2022 Jun 17;12:10259. doi: 10.1038/s41598-022-13766-1 (PMC9205868; doi:10.1038/s41598-022-13766-1)
Supplement: Supplementary file 8 — Supplementary Information 8. [file 41598_2022_13766_MOESM8_ESM.pdf]

GRUPOS	teste microdureza	VALORES	módulo de elasticidade
CONTROLE	31,33	10762	3,59
CONTROLE	35,10	10086	3,36
CONTROLE	36,10	11295	3,77
CONTROLE	36,10	10670	3,56
CONTROLE	36,97	10538	3,51
CONTROLE	35,70	9415	3,14
ARGILA 0.3	48,40	1,29	0,43
ARGILA 0.3	47,00	0,907	0,30
ARGILA 0.3	48,57	0,808	0,27
ARGILA 0.3	47,80	0,819	0,27
ARGILA 0.3	51,70	0,746	0,25
ARGILA 0.3	44,37	0,74	0,25
ARGILA 0.5	47,50	10,432	3,48
ARGILA 0.5	55,83	10,993	3,66
ARGILA 0.5	50,77	11,027	3,68
ARGILA 0.5	50,10	11,99	4,00
ARGILA 0.5	53,70	10,98	3,66
ARGILA 0.5	54,47	10,8	3,60
GRAFENO 0.3	52,63	8,42	2,81
GRAFENO 0.3	49,07	8,75	2,92
GRAFENO 0.3	55,83	8,22	2,74
GRAFENO 0.3	57,17	7,58	2,53
GRAFENO 0.3	57,93	7,15	2,38
GRAFENO 0.3	56,33	7,36	2,45
GRAFENO 0.5	54,40	3,3	1,10
GRAFENO 0.5	43,03	2,64	0,88
GRAFENO 0.5	46,13	4,74	1,58
GRAFENO 0.5	56,27	4,2	1,40
GRAFENO 0.5	46,90	2,57	0,86
GRAFENO 0.5	48,30	2,98	0,99
M1R 0.3	67,53	9,83	3,28
M1R 0.3	65,63	9,02	3,01
M1R 0.3	62,73	9,94	3,31
M1R 0.3	63,93	9,57	3,19
M1R 0.3	63,73	9,77	3,26
M1R 0.3	67,27	9,32	3,11
M1R 0.5	71	8,77	2,92
M1R 0.5	67	8,9	2,97
M1R 0.5	68	8,57	2,86
M1R 0.5	70	9,32	3,11
M1R 0.5	67	8,9	2,97
M1R 0.5	69	9,16	3,05
