# Supplementary material for: Enhancing the mechanical properties and providing bioactive potential for graphene oxide/montmorillonite hybrid dental resin composites
Source: Sci Rep. 2022 Jun 17;12:10259. doi: 10.1038/s41598-022-13766-1 (PMC9205868; doi:10.1038/s41598-022-13766-1)
Supplement: Supplementary file 9 — Supplementary Information 9. [file 41598_2022_13766_MOESM9_ESM.pdf]

ID	GRUPO	MODULO ELASTICIDADE
1	CONTROLE	3,617
2	CONTROLE	2,992
3	CONTROLE	3,973
4	CONTROLE	3,647
5	CONTROLE	2,936
6	CONTROLE	3,877
7	MONOMERO/PLA	3,023
8	MONOMERO/PLA	3,073
9	MONOMERO/PLA	3,047
10	MONOMERO/PLA	2,811
11	MONOMERO/PLA	3,039
12	MONOMERO/PLA	2,662
13	MONOMERO/PLA/GO 1	3,340
14	MONOMERO/PLA/GO 1	3,795
15	MONOMERO/PLA/GO 1	3,334
16	MONOMERO/PLA/GO 1	3,260
17	MONOMERO/PLA/GO 1	3,159
18	MONOMERO/PLA/GO 1	3,187
